# 4-1BB agonism: adding the accelerator to cancer immunotherapy

**DOI:** 10.1007/s00262-016-1829-2

**Published:** 2016-03-31

**Authors:** Cariad Chester, Siddhant Ambulkar, Holbrook E. Kohrt

**Affiliations:** 1 Department of Medicine, Division of Oncology, Stanford University Medical Center, Stanford University, 269 Campus Drive, CCSR 1140, Stanford, CA 94305-5151 USA; 2 Institute for Immunity, Transplantation and Infection, Stanford University School of Medicine, Stanford, CA 94305 USA

**Keywords:** CIMT 2015, 4-1BB, Immunotherapy, Antibody-dependent cell-mediated cytotoxicity, Combination therapy, Checkpoint inhibitors

## Abstract

The success of checkpoint inhibitors has validated immunomodulatory agents as a valuable class of anticancer therapeutics. A promising co-stimulatory immunologic target is 4-1BB, or CD137, a member of the tumor necrosis factor receptor superfamily. Ligation of 4-1BB induces an activating signal in CD8^+^ T cells and natural killer cells, resulting in increased pro-inflammatory cytokine secretion, cytolytic function, and antibody-dependent cell-mediated cytotoxicity. Targeting 4-1BB with agonistic monoclonal antibody (mAb) therapy demonstrated potent antitumor effects in murine tumor models. While anti-4-1BB mAbs have entered clinical trials, optimal efficacy of 4-1BB-targeted agents will inevitably come from combination therapeutic strategies. Checkpoint blockade is a compelling combination partner for 4-1BB agonism. This novel immunotherapeutic approach has the potential to active antitumor immune effectors by a complementary mechanism: simultaneously “removing the brakes” via blocking inhibitory signaling and “stepping on the accelerator” via co-stimulation. While important considerations should be given to 4-1BB-mediated toxicities, the current understanding of 4-1BB biology suggests it may play a key role in advancing the capabilities of cancer combination therapy.

## Introduction

4-1BB (CD137/TNFRSF9) was discovered in 1989 during screens for novel receptors on murine T-cell lines [1]. Using stimulation with concanavalin A (Con A), Kwon and Weissman identified 4-1BB as an inducible gene on T cells and successfully cloned the 4-1BB cDNA. In the last two decades, our understanding of the biology and function of 4-1BB has expanded tremendously. 4-1BB is now accepted as an activation-induced, co-stimulatory receptor that governs the function of diverse immune cell subsets. With the advent of cancer immunotherapy, targeting 4-1BB emerged as a promising therapeutic strategy. Analogously to checkpoint inhibitors “removing the brakes” on tumor-targeting effectors, 4-1BB co-stimulation may result in “pressing the accelerator,” or increasing immune effector cytotoxicity. Recently, two different monoclonal antibodies (mAb) targeting 4-1BB have entered clinical testing. In this review, we present the current understanding of 4-1BB expression and signaling and discuss the potential of 4-1BB mAb therapy to eradicate tumor and synergize with other therapeutic modalities.

## 4-1BB expression and signaling

The expression of 4-1BB has been reported on diverse immune cell populations. On T cells, natural killer (NK) cells, regulatory T cells (Treg), and NK T cells (NKT), 4-1BB expression is activation dependent [2]. Some of the canonic immunostimulatory agents known to induce 4-1BB upregulation on T cells include: Con A, plate-bound anti-CD3, CD28, phorbol myristoyl acetate (PMA), ionomycin, phytohemagglutinin, interleukin (IL)-2, or IL-4 [3, 4]. On NK cells, 4-1BB is upregulated following ligation of the FcRγIII Fc receptor (CD16) by the Fc portion of mAb. In addition to activated immune effectors, 4-1BB is also expressed on innate immune cell populations, including neutrophils, granulocytes, monocytes, mast cells, eosinophils, and dendritic cells (DC) [5]. Expression of 4-1BB has recently been shown on subtypes of lymphomas and leukemias, but its role in malignant transformation remains unclear [6, 7].

Once expressed, 4-1BB binds to a high-affinity 4-1BB ligand (4-1BBL or CD137L). 4-1BBL is predominantly expressed by activated antigen-presenting cells (APCs), including B cells, DCs, and macrophages [8]. Upon ligation and crosslinking, 4-1BB associates with the tumor necrosis factor (TNF)-associated factors 1 and 2 (TRAF1 and TRAF2) and activates the master immunoregulatory transcription factor NF-κB. In T cells, 4-1BB signaling upregulates the antiapoptotic B-cell lymphoma-extra large (Bcl-xl) and B-cell lymphoma 2 (Bcl-2) pathways and induces proliferation and production of pro-inflammatory cytokines interferon gamma (IFN-γ) and IL-2 [9, 10]. Furthermore, 4-1BB stimulation increases signaling through the T-cell receptor (TCR) and amplifies the cytotoxicity of CD8^+^ T cells [11]. Similarly, in NK cells, CD137 stimulation enhances proliferation, IFN-γ production, and cytolytic action [12]. In DCs, CD137 ligation accelerates maturation through upregulation of B7 co-stimulatory molecules (CD80 and CD86) and increases survival and production of IL-6 and IL-12 [13].

## 4-1BB in cancer immunotherapy

The initial evidence that anti-4-1BB agonistic mAbs may have strong antitumor effects emerged from studies on the highly tumorigenic P815 mastocytoma and poorly immunogenic Ag104A sarcoma [14]. Administration of anti-4-1BB mAb eradicated established tumors in both models and generated increased numbers of tumor-specific cytotoxic T cells. Importantly, anti-4-1BB mAb therapy appeared to trigger an immunologic memory response: Mice that had been cured of P815 tumor were challenged 3 months later with P815 cells and, after a period of transient growth, all tumors regressed. The antitumor capacity of 4-1BB-targeted mAb has since been replicated in a variety of tumor models, including lymphoma, hepatocellular carcinoma, and colon cancer [15–17]. The mechanism of action underpinning 4-1BB-mediated tumor regression consists of multiple, complimentary antitumor immune pathways. Primarily, 4-1BB agonism induces a potent, cytotoxic T-cell population that can infiltrate and lyse tumors [18]. In addition to direct tumor lysis, 4-1BB stimulation promotes the secretion of type 1 cytokines, creating an inflammatory, immunogenic cytokine milieu within the tumor microenvironment (TME) [19]. Finally, 4-1BB ligation increases the secretion of perforin and granzyme and activation of the Fas ligand (FasL) effector system by both CD8^+^ T cells and NK cells [20].

## 4-1BB stimulation enhanced ADCC

The role of antibody-dependent cell-mediated cytotoxicity (ADCC) is increasingly appreciated as an important component of antibody-mediated antitumor effects [21, 22]. In ADCC, Fc*γ*RIII (CD16) ligation by the Fc domain of immunoglobulins transmits activating signals within NK cells. In the cancer setting, tumor-targeted mAb is recognized by NK cell Fc*γ*RIII, triggering release of perforin and granzyme [23]. These direct cytotoxic effects are complemented by a release of interferon gamma (IFN-γ) which can recruit adaptive immune cells and promote antigen presentation by APCs [24]. Human NK cells upregulate 4-1BB after FcRIII ligation, and subsequent stimulation with anti-4-1BB mAb enhances ADCC. Enhanced ADCC-mediated tumor clearance makes anti-4-1BB antibodies an ideal candidate for therapeutic combinations with FDA-approved mAbs. We have previously demonstrated that antibodies targeting 4-1BB synergize with rituximab (anti-CD20) and trastuzumab (anti-HER2) to clear tumors in murine xenograft models of lymphoma and breast cancer [25, 26]. The synergistic therapeutic effect was dependent on Fc*γ*RIII expression and observed in mice competent in NK cells and macrophages, but lacking an adaptive immune system. Recently, we combined cetuximab (anti-EGFR) and anti-4-1BB antibody therapy to obtain complete tumor resolution and prolonged survival in models of colorectal cancer and head and neck cancer [27]. Based on the preclinical successes of antibodies targeting 4-1BB, two agonistic anti-4-1BB mAb entered clinical testing.

## 4-1BB clinical trials

The two anti-4-1BB mAbs currently in the clinic are urelumab (Bristol-Myers Squibb), a fully humanized IgG4 mAb, and PF-05082566 (Pfizer), a fully human IgG2 mAb (Table 1). Urelumab is now being tested in multiple combinatorial regimens: cetuximab in head and neck or colorectal cancer, rituximab in relapsed or refractory B-cell NHL, and elotuzumab (anti-SLAMF7) in multiple myeloma. PF-05082566 is currently in three clinical trials, and results of the phase I monotherapy study were recently reported [28]. Out of 27 treated patients, the best clinical response was disease stabilization, occurring in 22 % (6/27) of patients. Importantly, PF-05082566 did not induce any dose-limiting toxicities (DLTs), and all study discontinuations were from disease progression, not adverse events. Ongoing clinical studies test PF-05082566 in combination with rituximab and mogamulizumab (anti-CCR4).

**Table 1:** List of anti-4-1BB mAb clinical trials

NCT number^a^	Phase	Condition	Combination	Start year	Status (2014/12)
*Urelumab (BMS*-*663513)* ^*b*^ *: fully human type IgG4, Bristol*-*Myers Squibb*					
NCT00309023	I/II	Metastatic or locally advanced solid tumors	–	2005	Terminated
NCT00351325	I	Advanced solid malignancies	Chemotherapy	2007	Terminated
NCT00461110	I	Non-small cell lung cancer	Chemoradiation	2008	Terminated
NCT00612664	II	Melanoma	–	2008	Completed
NCT00803374	I	Advanced malignant melanoma	Ipilimumab (anti-CTLA-4 mAb)	2010	Withdrawn
NCT01471210	I	Advanced and/or metastatic solid tumors Relapsed/refractory B-cell non-Hodgkin’s lymphoma	–	2012	Recruiting
NCT01775631	Ib	Relapsed/refractory B-cell non-Hodgkin’s lymphoma	Rituximab (anti-CD20 mAb)	2013	Recruiting
NCT02110082	Ib	Colorectal cancer, head and neck cancer	Cetuximab (anti-EGFR mAb)	2014	Recruiting
NCT02252263	I	Multiple myeloma	Elotuzumab (anti-CS1 mAb)	2014	Recruiting
NCT02253992	I/II	Advanced solid tumors, advanced B-cell NHL	Nivolumab (anti-PD-1 mAb)	2014	Recruiting
*PF*-*05082566* ^*c*^ *: fully human type IgG2, Pfizer*					
NCT01307267	I	CD20 positive non-Hodgkin’s lymphoma	Rituximab (anti-CD20 mAb)	2011	Recruiting
NCT02179918	Ib	Advanced solid tumors	MK-3475 (anti-PD-1 mAb)	2014	Recruiting

^a^ *NCT* National Clinical Trial

^b^ *BMS* Bristol-Myers Squibb

^c^ *PF* Pfizer

As anti-4-1BB targeted agents advance in clinical trials, monitoring potential toxicities is a chief responsibility. Liver inflammation was observed during 4-1BB monotherapy, characterized by elevated levels of aspartate aminotransferase (AST) and alanine aminotransferase (ALT). Grade 2+ neutropenia, leukopenia, and thrombocytopenia were also observed [29]. Potentially fatal hepatitis was later observed in a Bristol-Myers Squibb (BMS) phase II anti-4-1BB study for previously treated stage III/IV melanoma, National Clinical Trial (NCT) 00612664. This study and several others (NCT00803374, NCT00309023, NCT00461110, NCT00351325) were terminated due to these adverse events. Despite this clinical hurdle, further studies have proven that hepatic toxicity due to 4-1BB agonist antibodies can be largely mitigated by lowering the dose of anti-4-1BB mAb administered.

## 4-1BB and checkpoint inhibitors

Checkpoint inhibitors are a novel and promising class of immunotherapeutic antibodies and have evoked impressive clinical responses to melanoma, non-small-cell lung cancer, and renal cancer. Immune checkpoints are inhibitory pathways that downregulate activated T cells following antigen presentation and co-stimulatory signaling by APCs. Currently, two checkpoint receptors have been targeted by FDA-approved mAbs: CTLA-4, targeted by ipilimumab, and programmed death (PD)-1. The recent approvals of PD-1 blocking mAb pembrolizumab and nivolumab validate the effectiveness of checkpoint blockade strategy in the setting of clinical oncology. Conceptually, the addition of 4-1BB agonists to a therapeutic backbone of checkpoint blockade is highly appealing. This combination targets two distinct elements in the process of immune effector activation: overcoming inhibition and co-stimulation. Checkpoint inhibitors can block the inhibitory signals that pervade the immunosuppressive TME, while 4-1BB stimulation can augment effector cells’ baseline cytotoxic capacity. If both molecules are simultaneously targeted, selective activation of tumor-targeting T and NK cells may occur, releasing a robust antitumor immune response. To date, there is strong clinical preclinical evidence to support this combination.

The combination of anti-CTLA-4 antibodies and anti-4-1BB has demonstrated preclinical success in multiple tumor histologies. In the B16 melanoma model, the combination showed a synergistic curative effect and induced antitumor TME remodeling; after therapy, the number of CD8^+^ T cells infiltrating the tumor increased, while the numbers of Tregs and myeloid-derived suppressor cells (MDSCs) decreased [30]. In the RM-1 prostate cancer model, a 4-1BBL-expressing tumor cell vaccine combined with CTLA-4 blockade caused tumor rejection in four out of five mice implanted with RM-1 tumors [31]. In the MC38 colon adenocarcinoma model, the combination of anti-4-1BB mAb and anti-CTLA-4 antibody led to the eradiation of large, established tumors (>7 mm in diameter) in seven out of eight mice and induced long-lasting immunity to rechallenge with MC38 cells [32]. More importantly than the observed synergistic therapeutic activity, Kocak et al. [32] demonstrated that combining anti-4-1BB with anti-CTLA-4 has the potential to ameliorate the toxicities observed with monotherapeutic use of anti-4-1BB mAb. Anti-CTLA-4 therapy reduced the 4-1BB-induced influx of T cell infiltrates into the liver and prevented the hepatic toxicity that has limited 4-1BB application in the clinic.

The PD-1 blocking mAbs nivolumab and pembrolizumab have demonstrated comparable therapeutic activity to ipilimumab while also having a milder toxicity profile. These observations suggest that PD-1 blockade may be superior to anti-CTLA-4 antibodies for combination with 4-1BB agonists. In one of the first preclinical models of this therapeutic strategy, Xiao et al. [33] combined soluble PD-1 with naked plasmid p4-1BBL-transfected H22 tumor cells in an ectopic hepatocarcinoma model. This combination demonstrated robust therapeutic synergy, increasing the number of infiltrating T cells and eradicating 60 % of established tumors. Recent testing of anti-4-1BB with PD-1 blockade further supports the argument for combinatorial synergy. In the CT26 colon adenocarcinoma model, combining 4-1BB agonism and anti-PD-1 mAb resulted in complete tumor rejection in all mice injected with CT26 cells [34]. In the poorly immunogenic B16-F10 melanoma model, combination therapy again resulted in complete tumor regression and an increased ratio of CD8^+^ T cells to Tregs [35]. However, Chen et al. [35] also demonstrated that the combination of anti-4-1BB and anti-PD-1 may exacerbate toxicities observed with anti-4-1BB monotherapy. At the higher dose levels of anti-4-1BB mAb (1 and 5 mg/kg), AST and ALT levels were elevated over twofold what was reported with anti-4-1BB monotherapy and the combination intensified anti-4-1BB-induced neutropenia, lymphopenia, and thrombocytopenia. These results suggest that PD-1 blockade may be a potent therapeutic partner for anti-4-1BB, but clinicians must progress cautiously with early-phase trials until an appropriate dosage is established.

Another approach to inhibiting the PD-1-mediated suppressive signaling is to block the ligand of PD-1 ligand (PD-L1). This approach has exhibited exciting early data, and currently, four clinical anti-PD-L1 mAbs are in testing: atezolizumab and MEDI4736 (both Fc null variants of human IgG1), MSB001078C (IgG1), and BMS-936559 (IgG4). In the ID-8 ovarian adenocarcinoma model, the combination of anti-PD-L1 and anti-4-1BB antibodies increased overall survival and enhanced T-cell effector function [36]. As anti-PD-L1 mAbs advance in clinical trials, combination therapy with anti-4-1BB mAbs will undoubtedly receive increased consideration. Currently, 4-1BB and anti-PD-1 checkpoint inhibitors are the focus of ongoing clinical trials. A phase I/II trial of urelumab with nivolumab in both solid tumors and B-cell non-Hodgkin’s lymphoma is recruiting participants (NCT02253992), while PF-05082566 is being tested in a phase Ib trial with pembrolizumab in patients with solid tumors (NCT02179918).

## 4-1BB and cancer vaccines

Recently, there has been a renewed focus on therapeutic cancer vaccines. The allure of vaccine strategies is the potential to “teach” individual patients’ immune systems to recognize, target, and eradicate tumor cells in an approach that employs both adaptive and innate immunity. Vaccines aim to provoke a tumor-specific immune response by increasing TAA presentation by APCs, thus generating tumor antigen-specific cytotoxic T lymphocytes. Unlike chemotherapy, surgery, radiotherapy, or antibody therapy, an effective vaccine-induced immune response could establish a state of immunologic memory that persists after tumor clearance and indefinitely suppresses tumor regrowth. Mechanistically, the combination of vaccine strategies and anti-4-1BB targeted agents is compelling because it targets synergistic and non-overlapping components of the antitumor immune response; vaccines generate an immune response and anti-4-1BB further activates tumor-targeted effector populations.

Ongoing efforts to combine cancer vaccines and anti-4-1BB agents have demonstrated early progress. In the HPV E6/E7-driven TC-1 murine cervical cancer model, vaccination with HPV E6/E7 peptides in conjunction with systemic 4-1BB agonistic antibody induced durable tumor regression in five out of eight mice. Surviving mice that were rechallenged with additional TC-1 cells demonstrated strong CD8^+^ T cell memory responses, remaining tumor free. Anti-4-1BB antibodies proved to be a superior combination partner for vaccine therapy, demonstrating superior results relative to CTLA-4 and PD-1 checkpoint inhibitors as well as other TNF family receptors [37]. In a MCA205 murine fibrosarcoma model, a tumor lysate-pulsed dendritic cell vaccine (TP-DC) combined with α4-1BB significantly reduced lung metastases. The combined therapy also led to regression of lung tumors in a B16-BL6 melanoma model [38].

## Conclusion

Significant progress has been made in recent years in elucidating the expression, function, and therapeutic potential of 4-1BB. The ongoing clinical trials dedicated to 4-1BB agonists portend the viability of 4-1BB as an important immunotherapeutic target. The maximal clinical efficacy of 4-1BB will emerge in combination treatment strategies, and checkpoint inhibitors are a compelling class of partner candidates. However, the broad range of 4-1BB expression makes off-target immune pathology an acute risk. Further research is needed to optimize approaches to harnessing 4-1BB-mediated antitumor effects, while minimizing auto-inflammatory complications. If this goal can be achieved, 4-1BB agonists may provide a critical component of future anticancer combination therapies.

## Abbreviations

ADCC: Antibody-dependent cell-mediated cytotoxicity
ALT: Alanine aminotransferase
AST: Aspartate aminotransferase
BMS: Bristol-Myers Squibb
DLT: Dose-limiting toxicity
NCT: National Clinical Trial
NKT: Natural killer T cell
PF: Pfizer
TME: Tumor microenvironment
Treg: Regulatory T cell
